# Correlation Analysis of Craniovertebral Angle and Scapular Index with Muscle Tone, Pulmonary Function, Balance Control, and Proprioception

**DOI:** 10.3390/life15101526

**Published:** 2025-09-27

**Authors:** Sang-Hun Jang, Sun-Wook Park, Seong-Gil Kim

**Affiliations:** 1 Department of Physical Therapy, College of Health and Life Science, Korea National University of Transportation, Jeungpyeong-gun 27909, Chungcheongbuk-do, Republic of Korea; upsh22@ut.ac.kr; 2 Department of Physical Therapy, College of Health Science, Kangwon National University, Samcheok-si 25949, Gangwon-do, Republic of Korea; swpt.park@kangwon.ac.kr

**Keywords:** posture, pulmonary function tests, muscle tonus, proprioception, postural balance

## Abstract

Forward head posture and altered scapular alignment are associated with musculoskeletal dysfunctions and impaired physical performance. However, the relationship between postural alignment indices and physiological function in young adults remains unexplored. A total of 54 healthy participants (mean age: 21.88 ± 2.06 years) were evaluated. Craniovertebral angle (CVA) and scapular index were measured as indicators of postural alignment. Upper trapezius muscle tone was assessed using the MyotonPRO device. Pulmonary function parameters, including the forced vital capacity (FVC) and forced expiratory volume in one second (FEV_1_)/FVC, were measured using spirometry. Balance control was evaluated using the Tetrax system, and cervical proprioception was assessed using joint position error (JPE) tests. CVA showed statistically significant but weak correlations with the muscle tone (r = −0.191), weight distribution index in the eyes-open condition (r = −0.199), and cervical flexion JPE (r = −0.198) and a positive correlation with FVC (r = 0.251) (*p* < 0.05). Scapular index showed a positive correlation with FEV_1_/FVC (r = 0.241) and a negative correlation with balance control (r = −0.213) (*p* < 0.05). Improved postural alignment, as reflected by a higher CVA and scapular index, was associated with reduced muscle tone, enhanced pulmonary function, better balance control, and more accurate cervical proprioception.

## 1. Introduction

In contemporary society, prolonged engagement in sedentary activities, such as smartphone use, computer work, and driving, has become pervasive, contributing to the widespread adoption of poor postural habits [1,2]. These behaviors impose continuous mechanical stresses on the cervical and thoracic spine, leading to various musculoskeletal disorders [3,4]. The rising prevalence of posture-related musculoskeletal dysfunctions has been associated with a diminished quality of life and performance impairments in activities of daily living [5].

Forward head posture (FHP) and altered scapular alignment are commonly observed in individuals with poor posture and can increase upper trapezius muscle tone and impair balance control [6]. Additionally, forward displacement of the cervical spine can restrict thoracic mobility, thereby adversely affecting pulmonary function, particularly the forced vital capacity (FVC), and reducing overall respiratory efficiency [7]. Postural misalignment has also been linked to diminished proprioceptive acuity, further compromising postural stability and motor control [8]. These physiological alterations not only result in short-term discomfort and dysfunction but may also contribute to chronic pain syndromes and a progressive decline in physical capabilities.

To objectively assess postural alignment, the craniovertebral angle (CVA) and scapular index are widely utilized in clinical and research settings [9,10]. The CVA quantifies the FHP by evaluating the anterior displacement of the head relative to the shoulder girdle, whereas the scapular index assesses the scapular alignment by measuring the distance between the medial border of the scapula and the thoracic vertebrae. These indices not only reflect structural postural deviations but also provide meaningful insights into associated functional impairments.

Despite their clinical utility, most previous studies examined these postural indices in isolation or in relation to a limited range of physiological variables. Comprehensive investigations into how the CVA and scapular index relate to muscle tone, fatigue, pulmonary function, balance control, and proprioception remain scarce [11]. Systematic analysis of these associations could enhance the diagnostic and prognostic value of postural alignment assessments in clinical practice. Furthermore, establishing robust correlations between alignment parameters and physiological functions may support the development of early intervention strategies and individualized rehabilitation protocols.

We hypothesized that greater CVA and Scapular Index values would be associated with lower muscle tone, better pulmonary function, improved balance control, and more accurate cervical proprioception.

Accordingly, the present study aimed to examine the correlations of the CVA and scapular index with key physiological functions, including muscle tone, fatigue, pulmonary function, balance control, and proprioception. These findings are expected to provide empirical evidence supporting the clinical application of postural alignment indices as effective tools for functional assessment and rehabilitation planning.

## 2. Materials and Methods

### 2.1. Participants

This study recruited 54 university students (48 males and 6 females) enrolled at S University in Chungcheongnam-do, South Korea. The mean age, height, and weight of the participants are presented in Table 1.

Based on prior studies, the minimum sample size required for correlation analysis was calculated using G*Power (version 3.1.9.7; Heinrich Heine University, Düsseldorf, Germany), with a significance level (α) of 0.05, statistical power (1–β) of 0.80, and effect size of 0.6 [12,13]. The minimum sample size was 44 participants. Therefore, 54 participants were enrolled to account for potential dropouts.

The inclusion criteria were as follows: Adults aged 20 years or older; No significant visual or somatosensory impairments that could affect participation; No current abdominal or low back pain that could interfere with the experiment; Not taking medications that affect muscle tone and a body mass index (BMI) < 30 kg/m^2^ [14]. Right-handed individuals only, to minimize variability due to limb dominance and potential postural asymmetry. Among the 54 participants, 48 were male and 6 were female, reflecting the gender distribution of the recruited university population; this imbalance is acknowledged as a limitation.

All participants provided written informed consent after receiving a thorough explanation of the study objectives and procedures. The study was performed in accordance with the ethical guidelines of the Declaration of Helsinki.

### 2.2. Balance Control Assessment

Balance control was evaluated using the Tetrax^®®^ posturography system (Sunlight Medical Ltd., Ramat Gan, Israel). This system consists of four independent force plates that assess vertical pressure changes and postural sway between the forefoot and heel regions, allowing the quantification of fall risk [15]. The participants stood barefoot on the device, facing forward.

Measurements were performed under two conditions: eyes open (EO) and eyes closed (EC). Each trial lasted 32 s, consistent with the device protocol and prior validation studies.

Participants were instructed to maintain their posture for 32 s under each condition. The device calculated two indices: the stability index and the weight distribution index (WDI). The stability index quantified postural sway, whereas the WDI reflected the distribution of weight across the anterior–posterior and medial-lateral directions. Lower values of both indices indicated better balance control [16].

### 2.3. Pulmonary Function Assessment

Pulmonary function was assessed using a portable spirometer (COSMED Srl, Albano Laziale, Italy). All procedures followed the technical standards of the American Thoracic Society (ATS) and European Respiratory Society (ERS) [17].

The participants wore nose clips and sat upright on a chair. After receiving verbal instructions and sufficient practice, the participants performed a maximal inhalation, followed by a rapid and forceful exhalation for approximately six seconds. The measured parameters included the forced expiratory volume in one second (FEV_1_), forced vital capacity (FVC), and their ratio (FEV_1_/FVC).

### 2.4. Muscle Tone Assessment

Muscle tone was measured in the nondominant (left) upper trapezius at the most tender trigger point using a handheld myotonometer (Myoton AS, Tallinn, Estonia) [18]. Participants sat in a neutral upright position with both hands resting on their thighs and looked straight ahead.

The left side was selected to ensure consistency across participants; prior studies have reported minimal side-to-side differences in resting trapezius tone, supporting this approach [18].

The most sensitive trigger point was identified and marked using a nontoxic washable ink pen. To minimize pre-measurement muscle activation, the participants rested for 5 min before data collection [19]. The probe was positioned perpendicular to the skin, and five mechanical impulses at a force of 0.4 N were applied, to quantify muscle tone in Hz. Each site was measured three times at 15 s intervals, and the mean value was used for analysis [20].

### 2.5. Craniovertebral Angle (CVA)

CVA was assessed as a quantitative indicator of forward head posture. Participants were seated comfortably in a natural position, and lateral-view photographs were taken. The images were analyzed using ImageJ software ver. 1.54j (National Institutes of Health, Maryland, USA). The angle was defined by the intersection between a line drawn from the C7 spinous process to the tragus of the ear and a horizontal reference line. A smaller angle reflects a greater anterior displacement of the head, indicating more severe forward head posture.

### 2.6. Scapular Index

The Scapular Index was calculated to examine the anteroposterior relationship between the scapula and thorax. It was determined using the following formula (Equation (1)):

(1) $$Scapular\;Index(\%)=\frac{Anterior\;Distance}{Posterior\;Distance}\;\times \;100$$

The anterior distance was measured from the midpoint of the sternal notch to the medial border of the coracoid process, while the posterior distance was defined as the distance from the postero-lateral tip of the acromion to the thoracic spinous process. This ratio reflects the positional balance of anterior and posterior structures. Lower values suggest increased postural deviation, such as rounded shoulders or thoracic kyphosis.

### 2.7. Statistical Analysis

Data were analyzed using SPSS for Windows (version 29.0; IBM Corp., Armonk, NY, USA). Descriptive statistics were used to summarize participants’ general characteristics (age, height, and weight).

Normality of the data was assessed using the Shapiro–Wilk test prior to correlation analysis. Pearson’s correlation coefficients were then calculated to examine associations of the CVA and Scapular Index with muscle tone, pulmonary function (FVC and FEV_1_/FVC), balance control, and proprioception. The significance level was set at α = 0.05.

## 3. Results

The CVA showed a significant negative correlation with the muscle tone (Hz) (r = −0.191) and a significant positive correlation with the FVC (r = 0.251) (*p* < 0.05, Table 2). Additionally, the CVA was negatively correlated with balance control ability, as measured by the weight distribution index in the eyes-open condition (WDI-EO; r = −0.199), and with proprioception, as indicated by the joint position error (JPE) during flexion (r = −0.198) (*p* < 0.05, Table 3 and Table 4).

The scapular index was positively correlated with the FEV_1_/FVC (r = 0.241) and negatively correlated with balance control ability (WDI-EO, r = −0.213) (*p* < 0.05; Table 2 and Table 3). Although statistically significant, all observed correlations were weak (r ≈ 0.2), suggesting limited clinical impact.

## 4. Discussion

This study analyzed the correlations between the CVA and scapular index—indicators of cervical and scapular alignment, respectively—and physiological function indicators such as muscle tone, pulmonary function, balance control, and proprioception. The results revealed that a greater CVA was associated with lower muscle tone and improved FVC, balance control (WDI-EO), and proprioceptive accuracy (JPE during flexion). In addition, a higher scapular index was positively correlated with the FEV_1_/FVC and negatively correlated with balance control. These findings suggest that postural alignment directly influences multiple physiological functions and support the clinical utility of function-oriented postural assessments. These findings should be interpreted as statistical associations rather than causal relationships, given the cross-sectional design. However, effect sizes were small (r ≈ 0.2), indicating limited clinical significance.

CVA quantifies the degree of anterior displacement of the head relative to the vertical axis of the body; a smaller angle indicates a more pronounced FHP [21,22]. Anatomically, FHP shifts the center of gravity of the head forward, increasing the load on cervical muscles such as the upper trapezius, levator scapulae, and scalene muscles [2]. These muscles must maintain continuous contraction to support the head, leading to metabolic waste accumulation and restricted blood flow, which, in turn, may result in increased muscle tone due to the heightened excitability of intramuscular proprioceptors [23]. The negative correlation between the CVA and muscle tone in the present study supports the idea that improved alignment reduces the functional overload in these muscles and physiologically alleviates muscle tone [18].

FHP also restricts thoracic movement and increases the reliance on accessory respiratory muscles. Malalignment limits diaphragmatic descent, inhibits upward rib cage movement, and restricts full thoracic expansion during inhalation. These restrictions can reduce both inspiratory and expiratory volumes, thereby diminishing pulmonary function, especially the FVC [24,25]. Conversely, improved cervical alignment, reflected by an increased CVA, facilitates thoracic expansion and improves the coordination between the diaphragm and intercostal muscles, resulting in an enhanced FVC [26]. This physiological mechanism explains the positive correlation between the CVA and FVC observed in this study.

From a sensory integration perspective, FHP may disturb the coordination of visual, vestibular, and somatosensory inputs [27,28]. Cervical alignment is closely linked to the proprioceptors in the upper cervical region, which contribute to postural stability through coordinated movements of the eyes, head, and neck. When the cervical spine protrudes forward, the tension in the deep cervical muscles (e.g., suboccipital muscles) increases, impairing the function of densely innervated muscle spindles and reducing the accuracy of the joint position sense. The negative correlation between the CVA and JPE-Flexion observed in this study may reflect the restoration of proprioceptive integration following improved alignment.

The association between postural alignment and balance control can be explained by the effect of alignment on the center of mass stability. FHP disrupts the anterior–posterior balance and increases the plantar pressure variability, compromising stable weight distribution [29]. An increased CVA indicates improved alignment, which allows the center of gravity of the head to realign with the vertical axis of the body, promoting a more even weight distribution and lowering the WDI-EO, thereby enhancing balance control.

The scapular index, which quantifies the position of the scapula relative to the thoracic spine, increases as the scapula becomes more stable and retracted. Because the scapula is a floating structure attached to the thoracic cage, its position significantly influences thoracic expansion and respiratory muscle mechanics. A low scapular index often indicates an anterior tilt and internal rotation of the scapula, which increases tension in the pectoralis major and minor, externally compresses the thoracic cavity, and restricts lung expansion. Conversely, a high scapular index supports effective thoracic expansion and improves the contractility of the diaphragm and external intercostals, potentially enhancing pulmonary indicators such as the FEV_1_/FVC [30].

In addition, the scapula plays a central role in upper limb motion and trunk stabilization, both of which are essential for balance control. Scapular asymmetry can disrupt static trunk balance and coordination between the upper and lower limbs, leading to abnormal weight distribution [31,32].

These findings are consistent with those of previous studies. Alaparthi et al. (2025) reported that FHP is associated with altered posture, pulmonary function, and impaired balance [33], whereas Yan, Zhou, and Kim (2025) demonstrated that combined scapular and respiratory training improves muscle activity and posture [34]. Kim and Kim (2019) further showed that oscillatory stimulation training of the shoulder joint influenced FHP angle and plantar pressure, supporting the relationship between cervical-scapular interventions and postural control [35]. Thongchote et al. (2024) also demonstrated that scapular stabilization exercises enhance respiratory muscle strength and chest mobility, contributing to balance control [31].

By demonstrating significant correlations between the CVA, scapular index, and physiological function, this study highlights the potential of these postural alignment indices as assessment tools and predictive markers for clinical intervention planning. These indicators may serve as valuable metrics in populations such as older adults at risk of falling, individuals with reduced pulmonary function, and patients with neuromuscular disorders exhibiting poor trunk stability. Moreover, the CVA and scapular index can be integrated into wearable sensor systems and digital health platforms, paving the way for function-alignment integrated evaluation tools. However, effect sizes were small (r ≈ 0.2), indicating limited clinical significance.

However, this study had several limitations. First, because the sample comprised healthy young adults, generalizing the findings to other age groups or clinical populations may be limited. Second, the cross-sectional design precludes causal inference, and the effect of alignment correction on function remains speculative. Third, effect sizes were small (r ≈ 0.2), indicating limited clinical interpretability. In addition, all measurements were performed by a single assessor, which may introduce potential measurement bias, and participants’ physical activity levels were not assessed, possibly confounding the observed associations. Finally, the marked gender imbalance (48 males, 6 females) and the homogeneous sample of Korean university students further restrict generalizability. Future studies should include larger and more diverse populations, employ multiple assessors, and consider activity-level stratification to improve generalizability and reproducibility.

## 5. Conclusions

This study investigated the relationship between postural alignment, specifically the CVA and scapular index, and various physiological functions. The findings demonstrated that better postural alignment is associated with lower muscle tone, enhanced pulmonary function, improved balance control, and more accurate proprioceptive acuity. These results suggest that the CVA and scapular index are not merely structural indicators but are meaningfully linked to broader aspects of physical function. These parameters may serve as exploratory indicators of posture-related physiological functions in young adults; however, longitudinal and interventional studies are required before firm clinical applications can be recommended.

Future studies should explore intergroup comparisons based on age and physical characteristics, apply diverse interventions, and conduct causal pathway analyses to elucidate the relationship between postural alignment and functional performance.

## Figures and Tables

**Table 1:** General subject characteristics (*n* = 54).

Variable	Mean ± SD
Age (year)	21.88 ± 2.06
Height (cm)	172.19 ± 5.15
Weight (kg)	73.56 ± 13.51
BMI (kg/m^2^)	24.52 ± 3.83

SD, standard deviation.

**Table 2:** Correlation of Craniovertebral Angle (CVA) and Scapular Index with Muscle Tone, Muscle Fatigue, FVC, and FEV1/FVC.

		Muscle Tone (Hz)	Muscle Fatigue (Log Decrement)	FVC (L)	FEV1/FVC (%)
		24.56 ± 1.43	1.03 ± 0.07	4.91 ± 1.08	76.83 ± 3.24
CVA (degree)	50.57 ± 1.14	−0.191 *	−0.144	0.251 **	−0.077
Scapular Index (%)	90.81 ± 3.63	0.139	0.137	0.127	0.241 *

Mean ± SD, * *p* < 0.05, ** *p* < 0.01, CVA, craniovertebral angle; FVC, forced vital capacity, FEV1, forced expiratory volume in 1 s.

**Table 3:** Correlations between Craniovertebral Angle (CVA) and Scapular Index with Eyes-Open and Eyes-Closed Weight Distribution Index (WDI) and Stability Score (ST).

		WDI-EO	WDI-EC	ST-EO	ST-EC
		3.66 ± 1.34	4.03 ± 1.27	15.55 ± 3.20	17.67 ± 2.60
CVA (degree)	50.57 ± 1.14	−0.199 *	−0.146	0.050	−0.051
Scapular Index (%)	90.81 ± 3.63	−0.213 *	−0.131	−0.136	−0.154

Mean ± SD, * *p* < 0.05, CVA, craniovertebral angle; WDI, Weight Distribution Index; ST, Stability Score; EO, Eye Open; EC, Eye Closed.

**Table 4:** Correlation between Craniovertebral Angle (CVA) and Scapular Index with Cervical Proprioceptive Sensations Measured by Joint Position Error(JPE) Tests.

		JPE-LtRot (Degree)	JPE-RtRot (Degree)	JPE-Flex (Degree)	JPE-Ext (Degree)
		4.27 ± 0.96	3.88 ± 0.89	4.17 ± 1.06	4.04 ± 1.04
CVA (degree)	50.57 ± 1.14	−0.011	−0.154	−0.198 *	−0.173
Scapular Index (%)	90.81 ± 3.63	−0.054	−0.132	−0.187	−0.162

Mean ± SD, * *p* < 0.05, CVA, craniovertebral angle; JPE, Joint Position Error Test; Lt, Left; Rt, Right; Rot, Rotation; Flex, Flexion; Ext, Extension.

## Data Availability

The data supporting the findings of this study are contained within the article. All values used in the analysis are presented in the tables of the manuscript.

## Abbreviations

The following abbreviations are used in this manuscript:

CVA	Craniovertebral angle
FVC	Forced vital capacity
FHP	Forward head posture
JPE	joint position error
